# Antibody Response to SARS-CoV-2 Vaccination in Patients With Lymphoproliferative Disorders and Plasma Cell Dyscrasias: Anti-Lymphoma Therapy as a Predictive Biomarker of Response to Vaccination

**DOI:** 10.3389/fonc.2022.840451

**Published:** 2022-07-07

**Authors:** Carol Gung, Regina McGuire, Mercy George, Abdullateef Abdulkareem, Katherine A. Belden, Pierluigi Porcu, Ubaldo Martinez-Outschoorn, Adam F. Binder, Inna Chervenova, Onder Alpdogan

**Affiliations:** ^1^ Division of Hematologic Malignancies, Department of Medical Oncology, Sidney Kimmel Medical College, Thomas Jefferson University, Philadelphia, PA, United States; ^2^ Sidney Kimmel Medical College, Thomas Jefferson University, Philadelphia, PA, United States; ^3^ Division of Infectious Diseases, Department of Medicine, Sidney Kimmel Medical College, Thomas Jefferson University, Philadelphia, PA, United States; ^4^ Division of Biostatistics, Sidney Kimmel Medical College, Thomas Jefferson University, Philadelphia, PA, United States

**Keywords:** vaccination, Covid-19, immune response, hematologic malignancies, chemotherapy.

## Abstract

We retrospectively analyzed SARS-CoV-2 vaccination antibody responses in a cohort of 273 patients with lymphoproliferative disorders or plasma cell dyscrasias who were seen at a single tertiary cancer center. Semi-quantitative anti-spike protein serologic testing was performed with enzyme immunoassay method. We found that the antibody response rate to SARS-CoV-2 vaccination was 74.7% in our patient cohort with no difference based on gender, age or race. The highest response rate was found in patients with Multiple Myeloma (MM) (95.5%). The response rates found in Diffuse Large B-Cell Lymphoma (DLBCL), Chronic Lymphocytic Leukemia (CLL), and Low-Grade Non-Hodgkin Lymphoma (LG-NHL) were 73.2%, 61.5% and 53% respectively. We also evaluated the effects of receiving active chemo-immunotherapy on SARS-CoV-2 vaccination antibody response. We found that the patients on treatment had lower response than the patients off treatment (62.1% versus 84.4% p<0.001). Thirty-four of 58 LG-NHL patients were receiving anti-lymphoma treatment with a lower SARS-CoV-2 vaccination response as compared to the patients who were not on treatment (29.4% v 87.5% p<0.001). We observed a similar pattern in CLL patients receiving treatment (48.1 v 76.0 p:0.049). We found that only disease type and treatment status (on-treatment vs. off- treatment), but not gender, age or race were significant predictors of non-response in the multivariable logistic regression model. The interaction between disease type and treatment status was not statistically significant by multivariate analysis. In conclusion, receiving anti-cancer treatment was found to play a significant role in decreasing the response to COVID-19 vaccination.

## Introduction

In the United States, two messenger RNA (mRNA) vaccines and an adenovirus vector-based vaccine have been granted Emergency Use Authorization from the Food and Drug Administration (FDA) for the prevention of the SARS-CoV-2 virus infection. The BNT162b2 (Pfizer/BioNTech) and the mRNA-1273 (Moderna) Covid-19 vaccines have both been shown in large phase III clinical trials to be more than 90 percent effective at preventing lab-confirmed Covid-19 illness and severe infections (1, 2). The single-dose recombinant, adenovirus based vaccine (Ad26.COV2.S; Johnson & Johnson/Janssen) reduced the incidence of symptomatic Covid-19 by 72% in the large phase III clinical trial (3). The Pfizer/BioNTech vaccine has subsequently been fully approved by FDA. These other two SARS-CoV-2 vaccines (Moderna and J&J/Janssen) are pending full FDA approval.

### Immune Response to mRNA Vaccine

Generally, mRNA vaccines deliver specific mRNA into host antigen presenting cells (such as dendritic cells). The majority of mRNA vaccines are packaged in biodegradable lipid nanoparticles (LNPs) consisting of phospholipids, cholesterol and polyethylene glycol (PEG) (4). This LNP package protects mRNA from degradation before translation. Vaccines that are mRNA based do not need to enter the nucleus to be effective and are not incorporated into the DNA. The mRNA vaccine interacts with ribosomes and stimulates viral protein production, which in this case is the Covid-19 spike protein. The specific protein generated by the vaccine mRNA is processed by the endoplasmic reticulum (ER) and proteasome system allowing viral peptides to be presented on the cell surface with MHC molecules. Antigen presentation leads to stimulation of T cells, especially helper T cells. Helper T cell expansion and activation stimulate cytotoxic T-cells (CD8+ T cells) and B cells. This process eventually stimulates germinal centers in the lymph nodes, which results in plasma cell proliferation and specific antibody production, which in this case is anti-spike protein immunoglobulin (4). Antibody levels against the spike protein can be measured in the serum to assess immune response to vaccination. Assessment of the T cell response to vaccination, another important component of immune protection, is not as easily measured.

### SARS-CoV-2 Vaccination in Immunocompromised Patients

People with immunocompromising conditions may be at increased risk of severe Covid-19. It is estimated that over 2% of the US population is immunocompromised (5). There are limited published data available to establish SARS-CoV-2 vaccine safety and efficacy in these groups. However, the current FDA-approved/authorized SARS-CoV-2 vaccines are not live vaccines and therefore can be safely administered to immunocompromised people. Patients with stable HIV infection were included in the SARS-CoV-2 vaccine clinical trials, although the number of participants were small. Reports of SARS-CoV-2 vaccine response in persons living with HIV have shown lower response rates as compared to the general population correlating with CD4+ count and degree of viral suppression*. A study with 658 solid organ transplant (SOT) recipients showed that only 98 (15%) of 658 subjects had a measurable antibody response after dose 1; 259 (39%) of 658 had no antibody response after dose 1 but subsequent antibody response after dose 2; and 301 (46%) of 658 had no antibody response after dose 1 or dose 2 (6). Assessments of a third full dose of SARS-CoV-2 vaccine in SOT recipients have shown improved response rates, albeit most notable in patients with an initial low level antibody response after either the first or second doses (7). The American Society of Transplantation now recommends a third full dose of mRNA vaccine in SOT recipients who have received two mRNA vaccine doses or a second dose of any vaccine in those who have received the J&J/Janssen vaccine (AST COVID-19 Vaccine FAQ Sheet. https://www.myast.org/sites/default/files/11.14.21-VaccineFAQ-Professionals.pdf). Additionally, recognizing that immunocompromised patients may not respond as well to a two shot series, the CDC recently recommended booster injection for adult patients 8 months after completion of their second dose of the Pfizer of Moderna vaccine.

Several studies have reported on SARS-CoV-2 vaccination response in patients with cancer. Monin et al. demonstrated poor antibody response after a single dose of the Pfizer mRNA vaccine in cancer patients (8). 151 patients with cancer (95 patients with solid cancers and 56 patients with hematological cancers) and 54 healthy controls were enrolled in the study. Positive anti-S IgG titers at approximately 21 days following a single vaccine inoculum across the three cohorts were 32 (94%; 95% CI 81-98) of 34 healthy controls; 21 (38%; 26-51) of 56 patients with solid cancers, and eight (18%; 10-32) of 44 patients with hematological cancers. The response rate improved after the second vaccination dose, which was given 21 days after the first. Another study from the University of Pittsburgh showed that 46% of patients with hematologic malignancies did not produce antibodies after two doses of the mRNA vaccines (9). Patients with Chronic Lymphocytic Leukemia (CLL) were at a particularly high risk of not mounting an antibody response to vaccination, as only 23% had detectable antibodies despite the fact that nearly 70% of these patients were not on cancer therapy. Van Okelen and colleagues reported that most of fully immunized patients with multiple myeloma mounted measurable SARS-CoV-2 spike-binding IgG antibody levels (84.2% (219/260)) (10). Moreover, patients receiving myeloma treatment had significantly lower SARS-CoV-2 spike-binding IgG antibody levels after vaccination compared to patients not receiving anti-myeloma therapy.

A recent study also evaluated SARS-CoV-2 vaccination response in a large group of 1400 patients with hematological malignancies from a prospective cohort registry study from March to May 2021. Antibody levels were tested at a median time of 42 days after receiving the 2^nd^ vaccine (11). They found that 75% of all patients with hematologic malignancies produced antibodies to the SARS-CoV-2 mRNA vaccines. Patients with the most common B cell malignancies had the lowest rate of seropositivity.

In sum, several studies have shown that patients with immunosuppressive comorbidities may mount suboptimal antibody responses to SARS-CoV-2 immunization. This was seen in cancer patients who are immunocompromised due to defects in humoral and cellular immunity due to the underlying malignancy as well as due to immunosuppressive therapy.

We assessed the antibody response to SARS-CoV-2 vaccination in our patients with lymphoproliferative disorders and plasma cell dyscrasias to further evaluate this high-risk population with impaired responses to other vaccines. We also evaluated the effects of anti-cancer treatment on the response to SARS-CoV-2 vaccination.

*: *The study, “Lower SARS-CoV-2 IgG and pseudovirus neutralization titers post-mRNA vaccination among people living with HIV,” was presented virtually at IDWeek 2021, held September 29-October 3, 2021.*

## Materials and Methods

### Study Characteristics

This is a retrospective cohort study. We aimed to evaluate Covid-19 vaccination response in patients with hematologic malignancies including lymphoproliferative disorders and plasma cell dyscrasias.

### Patient Population

We retrospectively analyzed adult patients who are older than 18 years old with lymphoproliferative disorders or plasma cell dyscrasias seen in our tertiary care cancer center from January 1, 2021 to August 10, 2021. We collected data including age, sex, cancer diagnosis, treatments, prior Covid-19 infections, vaccine type and dates of administration. The antibody response was assessed a minimum of 2 weeks after the second dose of the mRNA vaccines or 3 weeks from the one dose of the J&J vaccine.

Off-treatment and on-treatment: The patients who have not received any treatment for at least the last 3 months, are accepted as off-treatment status. If the patients who are actively receiving treatment or have received in the last 3 months, are accepted as on-treatment status.

### Covid-19 Antibody Test

Semi-quantitative anti-spike (S) serologic testing was performed with the Roche Elecsys anti-SARS-CoV-2 S enzyme immunoassay, which tests for the receptor-binding domain of S and correlates with neutralizing immunity mediated by vaccination (12). The sensitivity and specificity of the immunoassay is close to 100% for the detection of spike antibodies in response to Covid-19 vaccine.

Roche Diagnostic reported that Elecsys Anti Sars-Cov-2 S assay positive 225 of 233 samples were determined with > 0.8 U/ml considered positive, resulting in a positive percent agreement (PPA) of 99.6. (95% CI: 93.95-98.51) in the cohort. Specificity is reported 99.80 (95% CI 99.69-99.88%). Sensitivity was found as 85.3 (78.6 -90.6) 7-13 days after PCR confirmation and 99.5% (CI 97.0-100%) 14 days after PCR confirmation. Source: Roche Diagnostic

https://diagnostics.roche.com/us/en/products/params/elecsys-anti-sars-cov-2.html. We used 0.4 U/ml as cut-off for antibody response per our laboratory.

Patients positive for the anti-nucleocapsid antibody indicating a prior exposure to SARS-CoV-2, were included for the data analysis as long as they had completed the vaccination schedule for SARS-CoV-2.

### Statistical Analysis

The proportion of patients who developed a positive antibody response to the anti-S antibody was compared between patient characteristic groups and disease and treatments categories using the Fisher exact test and its extension (for more than 2 groups). All tests were two-sided, with α = 0.05. The multivariable logistic regression model was used to model the odds of non-response as dependent on patient characteristic groups and disease and treatments categories. Non-response was selected as outcome for the logistic regression model because the response was more prevalent than non-response. Disease type, gender, age group (<65 vs. 65+), race (dichotomized as white vs. non-white), and treatment status (on- treatment vs. off- treatment) were considered as predictors of non-response. The interaction between disease type and treatment status was also considered. The final model includes only significant (at the level 0.05) predictors. Statistical analyses were performed in R (R Core Team (2021). R: A language and environment for statistical computing. R Foundation for Statistical Computing, Vienna, Austria. URL https://www.R-project.org/.).

The study was approved by the Institutional Review Board of Thomas Jefferson University.

## Results

We retrospectively analyzed a cohort of 273 patients with a diagnosis of a lymphoproliferative disorder or plasma cell dyscrasia who had been seen at the Sidney Kimmel Cancer Center of Thomas Jefferson University Hospital from January 1 to August 10, 2021. 93 female and 180 male patients were enrolled with a median age of 67 years (27-94). Total response rate to SARS-CoV-2 vaccination was 74.7%. Sixty-nine of these patients did not have increased titers (25.3%). The age does not affect the Covid-19 vaccination response. There was no significant difference in vaccine response between patients older than 65 and those younger than 65 years (p=057). Response rates also did not differ on the basis of gender, with 71.8% response in males and 81.7% response in females (p=0.058) (**Table 1**).

**Table 1:** The response rates to Covid-19 vaccination.

Characteristics	Response	%	P Value
Gender			
Female	76/93	81.7	
Male	128/180	71.1	0.058
Age			
<65	86/112	76.8	
>=65	118/161	73.3	0.572
Treatment			
On treatment	74/119	62.1	
Off treatment	130/154	84.4	<0.001
RACE			
White	159/212	75.0	0.868 (*)
NonWhite	45/61	73.8	
AA	36/46	78.3	
Hispanic	4/5	80.0	
Asian	5/10	50.0	

(*) comparing response rates between non-White and White.

### Race

This cohort included Caucasian patients [212 patients (78%)], African American-AA patients (46 patients, 16.8%), hispanic patients (5 patients, 1.8%) and Asian American (10 patients, 2.9%). There was no significant difference in SARS-CoV-2 vaccination antibody responses in Caucasian versus African American patients (75%, versus 78.3% respectively, p>0.05) or between the Caucasian group compared to all patients belonging to minority groups (75% v 73.8% respectively; p=0.86)).

### Type of the Vaccine

Vaccine type was available for 267 patients. Seventy-nine patients received the Moderna vaccine of whom 69 had a response (83.5%). Looking at the overall response to the Pfizer vaccine, out of a total of 179 patients, 131 patients (73.2%) had a response. Six patients received the J&J vaccine of which 83.4% achieved a response. Moderna vaccine response was higher than Pfizer vaccine response, but was not statistically significant (p: 0.06, Relative risk: 1.15. (CI: 0.98 to 1.30)

### Intervals From the Vaccination

The median duration between two vaccinations is three weeks for Pfizer/BioNTech and four weeks for Moderna as recommended. We evaluated the interval between the last Covid-19 vaccination and the sample collection for antibody response. We found that the responders had a longer duration than the non-seroconverters (91.5 ± 6.5 versus 77.4 ± 9.6 days, P< 0.05). Therefore, the longer interval from the last vaccination did not affect the response to the covid-19 vaccine in our study.

### Subtype of Disease

We evaluated patients in five different disease categories including; i) chronic lymphocytic leukemia (CLL), ii) low grade B cell lymphoma-LG-NHL (follicular lymphoma (FL), marginal zone lymphoma, and Waldenstrom macroglobulinemia etc.), iii) diffuse large B cell lymphoma (DLBCL), iv) multiple myeloma (MM) and plasma cell dyscrasias, v) remaining other patients (OP). The details of the groups are given as follows

#### Multiple Myeloma/Plasma Cell Dyscrasias Group

Consists of 41 patients with multiple myeloma, 12 patients with MGUS, five patients with smoldering myeloma (SM), five patients with amyloidosis, and three patients with plasmacytoma. Eighteen patients underwent autologous stem cell transplantation. One patient had a transplant in the last six months prior to vaccination. Five of them had a transplant in the last 12 months prior to vaccination. Most of the transplanted patients have been on maintenance treatment, including lenalidomide (8/18, 44%), bortezomib (3/18, 16.6%), and daratumumab (2/18, 11.1%). As expected, most myeloma patients were on active treatment. The patients with MGUS, SM, and amyloidosis were off treatment.

#### DLBCL Group

We have 41 patients in this group. Six patients are on active treatment. Six patients have completed treatment in the last six months prior to vaccination. 23 of 41 (56%) patients did not have any treatment in the last 12 months. Three patients completed treatment in the last 12 months. Only 3 patients underwent autologous stem cell transplantation. One of them had a transplant in the last 12 months. The other two patients had a transplant greater than one year prior to vaccination.

#### LG-NHL

This group includes patients with follicular lymphoma, marginal zone lymphoma, Waldenstrom macroglobulinemia, and mantle cell lymphoma. Thirty-four patients were on treatment. Two patients completed the chemotherapy (R-CHOP) in the last six months. Two patients had autologous SCT, and one of them was performed in the last 12 months. The patients receive multiple chemotherapies in combination with anti-CD20 antibodies. Eleven of 34 (32.3%) patients receive a combination of ibrutinib and obinutuzumab. In addition, rituximab has been given to eight patients as a single agent (8/34, 23.5%). Twent-four patients were off treatment.

#### CLL Group

Fifty-two patients are in this group. Twenty-seven patients received treatment, including ibrutinib 10/27 (37%). Obinutuzumab and venetoclax combination is the second most frequently used regimen for patients with CLL (6/27, 22%). Twenty-five patients were off treatment. None of the CLL patients underwent stem cell transplant or cellular therapy in the last twelve months.

The response rates to SARS-CoV-2 vaccination are shown in **Table 2**. The highest response rate was found in patients with MM (95.5%). Response rates were 73.2% in DLBCL, 53.4% in low-grade lymphoma, 61.5% in CLL and 86% in OP. When we compared the groups, we found that the multiple myeloma group showed a better response than all other groups. There was a trend in the DLBCL group to have an increased antibody response rate compared to the LG-NHL group, but it was not statistically significant.(p=0.059).

**Table 2:** The response rates to Covid-19 vaccination according to disease.

Disease	Response (+)	%	Total
DLBCL	30	73.2**	41
LG-NHL	31	53.4	58
Myeloma	63	95.5	66
CLL	32	61.5	52
Other*	48	85.7	56
Total	204	74.7%	273

*Other: Hodgkin’s lymphoma, peripheral T cell lymphoma (PTCL), large granular lymphocytosis (LGL), lymphoproliferative disease, cutaneous T cell lymphoma (CTCL), Post-transplant lymphoproliferative disease (PTLD), Castleman disease etc.

**The overall difference in response rate is significant (p<0.001), showing differences in response rates between disease groups.

### Effects of Treatment

We found that receiving active chemo-and/or immunotherapy was associated with an impaired SARS-CoV-2 vaccination response especially in low-grade lymphoma and CLL patients. Thirty-four of 58 LG-NHL patients had received anti-lymphoma treatment including rituximab, obinutuzumab alone or in combination with chemotherapy, or Bruton Tyrosine Kinase (BTK) inhibitors at the time of their vaccinations. If the patients have received treatment in the last three months before the vaccination, in addition to the patients who are actively on treatment, they were accepted as active treatment. The antibody response to SARS-CoV-2 vaccination in patients receiving therapy was 10 of 34 patients (29.4%). The patients who were not on any treatment showed a significantly better response to vaccination (87.5%) compared to the patients who were receiving treatment (p<0.001), which is shown in **Table 3**. We observed a similar pattern in CLL patients. Thirteen of 27 patients with CLL that received treatment have an antibody response to Covid-19 vaccination (48%). The response rate was significantly higher at 76% in untreated CLL patients (p=0.049). However, we did not find a statistically significant difference in the multivariate analysis model in disease-specific groups. Overall, receiving treatment significantly affects to response to Covid-19 vaccination.

**Table 3:** The COVID-19 response rate by disease group on/off treatment.

Disease	Response (+)	%	Total	p-value (#)
CLL-off treatment*	19	76.0%	25	0.049
CLL on treatment	13	48.1%	27	
Myeloma off treatment*	26	92.9%	28	0.570
Myeloma on-treatment	37	97.4%	38	
DLBCL off treatment*	26	74.2%	35	0.65
DLBCL on-treatment	4	66.6%	6	
LG-NHL-off treatment*	21	87.5%	24	0.001
LG-NHL-on treatment	10	29.4%	34	
Other off treatment*	39	92.9%	42	0.018
Other on-treatment	9	64.3%	14	

*Testing the patients who have not received any treatment for at least in the last 3 months.

^#^Testing the difference between off treatment and on treatment response rates in each disease group.

**Figure 1:**
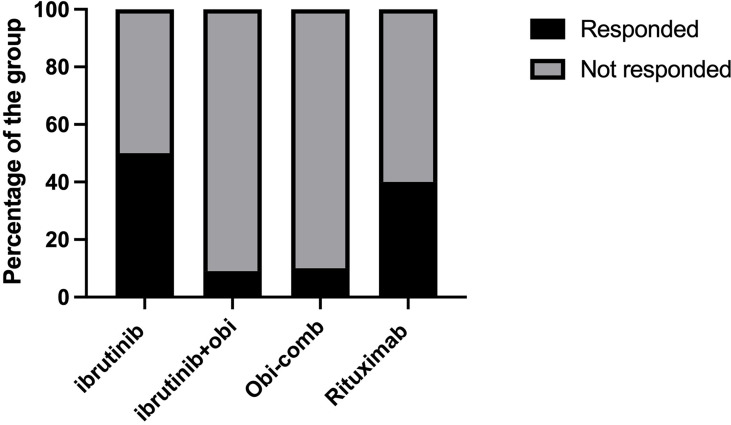
Effects of anti-cancer treatment on SARS-CoV-2 vaccination response. Obi, Obinutuzumab; Obi-Comb, Obinutuzumab+Venetoclax or Obinituzumab+venetoclax+ibrutinib.

We also found that the type of anti-cancer treatment was associated with response to SARS-CoV-2 vaccination. We combined both the CLL and LG-NHL groups to study treatment effects. BTK inhibitors in CLL and LG-NHL moderately decreased antibody responses to vaccination. Out of 110 patients in these 2 groups, 61 patients were on active anti-cancer treatment. Fourteen patients, who were on ibrutinib alone had a 50% response rate to the Covid-19 vaccination, which was similar to the overall CLL/LG-NHL group (57%). The response rate in the treatment group was only 38%, which was statistically significant different from the non-treatment group (81.6%, P<0.001). Interestingly, the patients who had received ibrutinib and obinutuzumab combination had only a 9.1% response rate to the SARS-CoV-2 vaccination (**Figure 1**), which is significantly lower than the ibrutinib alone group. Other patients with obinutuzumab combinations had a similar response rate (10%). The patients who received rituximab or rituximab containing combinations showed a trend towards higher responses to vaccination (40%), than patients with obinutuzumab containing protocols (9.5%). Interestingly, 5 patients who had been receiving only IVIG, responded well to COVID-19 vaccination (100%).

### Results From the Multivariable Logistic Regression Model for NON-Response

Considered jointly, only disease type and treatment status (on-treatment vs. off- treatment), but not gender, age group or race were significant predictors of non-response in the multivariable logistic regression model. In comparison to Myeloma patients with the highest response rate (**Table 2**), all other disease types had significantly higher odds of non-response (**Table 4**). That is, the odds of non-response were estimated to be 16.3 times higher for CLL (95%CI: 5.0-74.8; p<0.001), 16.6 times higher for DLBCL (95%CI: 4.4-82.9; p<0.001), 21.4 times higher for LGL (95%CI: 6.7-96.7; p<0.001), and 5.9 times higher for other category (95%CI: 1.5-29.0; p=0.014). The odds of non-response were 4.2 times higher (95%CI: 2.2-8.2; p<0.001) on-treatment as compared to off- treatment. The interaction between disease type and treatment status was not statistically significant, but the overall effect of treatment status appears to be important in CLL, LGL and other disease types (**Table 3**).

**Table 4:** Results from the multivariable logistic regression model for NON-response to Covid-19 vaccination.

	Odds	95% confidence interval		
Comparison	Ratio	Lower Limit	Upper Limit	p-value
CLL vs. Myeloma	16.3	5.0	74.8	<0.001
DLBCL vs. Myeloma	16.6	4.4	82.9	<0.001
LGL vs. Myeloma	21.4	6.7	96.7	<0.001
Other vs. Myeloma	5.9	1.5	29.0	0.014
On-Tx vs. Off-Tx	4.2	2.2	8.2	<0.001

### The Effects of Immunoglobulin Levels and Absolute Lymphocyte Count

We first evaluated the patients with low-grade lymphoma and CLL. We routinely check immunoglobulin levels in this patient population. The patients with Waldenstrom Macroglobulinemia are excluded from the analysis. Seventeen patients with LG-NHL or CLL (17 of 102 patients) had hypogammaglobulinemia (all three Immunoglobulins (IG) IgG, IgA, and IgM levels were lower than normal levels) prior to the vaccination. Their response to the vaccination is 35.2%, lower than the remaining LG-NHL/CLL patients’ response (61%, P: 0.06, relative risk 0.57 (CI: 0.27-0.99).

We then look at the myeloma group. We found that nine patients with hypogammaglobulinemia had decreased response. Since most of the patients have responded well to vaccination, we have looked at titers of antibody response in the myeloma patients. We found that there is no difference in the risk of developing hypogammaglobulinemia between patients with high titers 6/34 (17.6%) and the patients with low titers 3/19 (15.7%) (p>0.05)*. We then evaluated non-paraprotein immunoglobulin (IG) levels in the myeloma group. We found a reduction in non-paraprotein IG levels in 37 patients (non-paraprotein IG group). The chance of developing antibody response with high titers is similar in the non-paraprotein IG group and the patients with normal IG (68.75 *versus* 60.0% P>0.05).

(* If antibody titer against the SARS-CoV-2 spike protein is reported as higher than the maximum limits of laboratory values, it is accepted as high-titer. Remaining positive titers are accepted as low titers.)

We also compared absolute lymphocyte count (ALC) levels between the two groups and found no difference in the high and low titer groups (1389 ±105/mm3 versus 1266 ±152, respectively, P>0.05). Interestingly, ALC levels are similar in the LG-NHL and myeloma groups (1332 ± 99 versus 1345 ±100)

## Discussion

In this retrospective analysis, we evaluated 273 patients with lymphoproliferative disorders and plasma cell dyscrasias and observed that they had a decreased response to SARS-CoV-2 vaccination especially those with CLL and LG-NHL who had been receiving anti-cancer therapy. We consider that receiving anti-CD20 antibody results in a decreased antibody response to vaccination.

Anti-CD20 antibody treatment decreases the immune function. B cell count rapidly declines after anti-CD20 antibody (rituximab) therapy and slowly recovers over six months and requires approximately one year for complete restoration (13). Rituximab monotherapy did not significantly affect on CD3+, CD4+, and CD8+ T-cell counts. Anolik et al. reported a sustained increase in the percentage of transitional B cells and a slow increase in the number of memory B cells, which remained at very low levels even at one year after rituximab treatment (14). Rituximab use also decreases immunoglobulin production. The MSKCC lymphoma group evaluated serum IgG levels before and after rituximab therapy. After rituximab therapy, hypogammaglobulinemia was identified in 39% of patients with initially normal serum IgG levels (15). Symptomatic hypogammaglobulinemia, including sinopulmonary infections, prompted a start of IVIG administrations in 6.6% of the patients. Makatsori et al. from the UK found significant hypogammaglobulinemia in patients treated with rituximab. All patients had reduced or absent B-cells after anti-CD20 therapy. Haemophilus Influenzae B, tetanus and pneumococcal serotype-specific antibody levels were all reduced, and patients failed to mount an immune response post-vaccination (16).

Treatment with an anti-CD20 monoclonal antibody has negatively affected antibody response after influenza vaccination in lymphoma patients. Yri et al. reported that lymphoma patients who are undergoing treatment with rituximab-containing regimens or have received such regimens within the past six months were unable to mount protective antibody responses to the influenza A (H1N1) 2009 virus vaccine (Pandemrix) during the 2009 “swine flu” pandemic (17). Sixty-seven lymphoma patients and 51 healthy controls were enrolled in their study. Although 82% of the control group responded adequately to the vaccine, none of the 67 patients in the treatment arm achieved protective antibody titers. The median value of immunoglobulins, CD3^+^, CD4^+^, and CD8^+^ cell counts were within normal ranges in the patients. CD19^+^ B cells were undetectable in most of patients in the lymphoma group. We should consider that anti-CD20 treated patients might fail to respond not only to the influenza vaccine but also to other vaccines, including the Covid-19 vaccines.

Houot et al. showed negative effects of anti-CD20 antibodies on vaccine efficacy before the development of SARS-CoV-2 vaccines (18). Our study suggests that obinutuzumab may more profoundly decrease the SARS-CoV-2 vaccine response in patients with LG-NHL or CLL. Care needs to be exercised in using anti-CD20 antibodies during pandemics and alternative treatment options may need to be considered.

Gurion and colleagues reported the importance of timing of vaccination after anti-CD20 treatment to generate an antibody response to SARS-CoV-2 vaccination. They found that the antibody response to the BNT162b2 vaccine is reduced in lymphoma patients within the first 12 months following treatment with anti-CD20 antibodies (19). Other groups have also studied the effects of time from last anti-CD20 infusion to vaccination. The antibody response was improved if vaccinated greater than 9 months after completion of anti-CD20 therapy. In a cohort of four lymphoma subgroups, which included patients on active treatment or within 3 months after completion of treatment, patients that were 3 to 6 months post-anti-CD20 treatment, patients between 6 and 9 months post-anti-CD20 treatment, and patients that were more than 9 months after CD20–directed therapy, it was found that none of the patients demonstrated a significant IgM response to the vaccination (20). The IgG response in patients with ongoing treatment or vaccinated within 3 months from the last treatment was significantly lower than patients vaccinated more than 9 months after the last treatment. Their study suggested that SARS-CoV-2 vaccination at least 9 months from the last B cell-directed treatment might result in improved antibody titers. Despite these findings, the optimal timing of vaccine administration post treatment is unknown. While some have postulated that patients may still benefit from cellular immunity in the absence of humoral immunity, recent studies have demonstrated that these two immune responses tend to correlate well in terms of response to the COVID-19 mRNA vaccines (21). Taken together these studies suggest we should wait 6-9 months post B-cell directed therapy to get vaccinated, but further data with larger cohorts of patients is still needed to confirm these preliminary results.

Marchesi et al. found that patients receiving the SARS-CoV2 vaccines during treatment with anti-CD20 agents did not mount an antibody response to the vaccine. However some patients started to mount an antibody response if vaccinated at least 3 months after completion of anti-CD20 therapy (22). In our study, if the patient receives anti-CD20 antibody infusion in 3 months before the Covid-19 vaccination, is included to active treatment group.

A study by Greenberger et al. showed similar findings to our study in terms of a decreased vaccine response in lymphoma patients (11). The investigators evaluated leukemia, lymphoma and myeloma patients from a prospective cohort registry study from March to May 2021. Antibody levels were tested at a median time of 42 after receiving the 2^nd^ vaccine. In patients with B-cell malignancies, seronegativity was observed in almost all non-Hodgkin Lymphoma subtypes while all but one of 64 Hodgkin lymphoma patients were seropositive. Their analysis showed that patients with the most common B cell malignancies including CLL, FL, DLBCL and mantle cell lymphoma have the lowest rate of seropositivity (44% - 79%). Interestingly, we have not found the same pattern of reduced antibody responses in patients with multiple myeloma (MM) on anti-cancer treatment that we observed in LG-NHL and CLL patients receiving anti-cancer therapy. Eighteen out of 67 MM patients were on Lenalidomide and/or Bortezomib and all responded to SARS-CoV-2 vaccination. Hence, patients with MM on lenalidomide and/or bortezomib treatment had good antibody responses to SARS-CoV-2 vaccination in our study. Similarly, the study published by Greenberger et al. reported that 95.1% of myeloma patients responded well to Covid-19 vaccination (11). A recent study published in Cancer Cell, showed a slightly lower SARS-CoV-2 vaccination response in MM patients compared to our study. 320 MM patients were tested for an antibody response 10 days after the 2^nd^ vaccine dose of the Pfizer or Moderna vaccines and 84.2% of the patients were found to have a response after vaccination (10). Furthermore, they found that 58.5% of non-responders were on anti-CD38 antibody-containing therapy at the time of vaccination, 31.7% were on anti-BCMA bispecific antibody therapy, and 9.8% had undergone anti-BCMA CAR-T therapy more than three months prior. The study showed that MM patients receiving anti-CD38 or BCMA-targeted therapies had lower antibody levels.

Multiple myeloma is commonly associated with reducing of the serum levels of polyclonal immunoglobulins and the failure to synthesize a suitable antibody response following immunization. Immunoparesis is defined as reducing at least one uninvolved immunoglobulin level below the normal levels in patients with myeloma/plasma cell dyscrasias (23). Our data showed that immunoparesis does not affect antibody response after Covid-19 vaccination. The effect of immunoparesis on the outcome of myeloma is not clearly documented, which may impact through a combination of being associated with more aggressive disease and reduced immune surveillance of myeloma (24). On the other hand immunoglobulin replacement therapy appears to be one of the reasonable approaches for the treatment of immunodeficiency in patients with myeloma. Vacca et al. administered subcutaneous IVIg administration to prevent infectious complication in patients with myeloma in randomized clinical trial (25). It might be an alternative approach for patients who do not respond well to Covid-19 vaccination.

We consider that the study has several limitations, including the retrospective nature, no assessment of cellular responses, and lack of information on cumulative exposure and duration of corticosteroids and immunomodulators.

### Conclusion

Patients with lymphoproliferative disorders and plasma cell dyscrasias often have an inadequate antibody response to SARS-CoV-2 vaccination. Specifically, patients with B cell lymphoma receiving anti-CD20 antibodies had the lowest antibody responses in our cohort. Future studies will need to investigate whether administering SARS-CoV-2 vaccines the start of anti-CD20 therapy or postponing vaccination until completion of anti-CD20 antibody therapies improves antibody responses to SARS-CoV-2 vaccination. Assessment of vaccine response after booster and increasing vaccine dose in this high-risk patient population may be indicated. Other options to consider in patients with low-grade B cell lymphomas and CLL are to discuss the risks and benefits of using anti-CD20 antibody therapies and the administration of prophylactic passive immunization with anti-SARS-CoV-2 monoclonal antibodies. The efficacy of Covid-19 vaccination may affect the outcome of Covid-19 infection, the rate of hospital admission due to Covid-19 and the overall survival of the patients, which will be investigated in the larger prospective clinical studies.

## Data Availability Statement

The original contributions presented in the study are included in the article/supplementary material. Further inquiries can be directed to the corresponding author.

## Ethics Statement

The studies involving human participants were reviewed and approved by Institutional Review Board of Thomas Jefferson University, Philadelphia, PA, United States. Written informed consent for participation was not required for this study in accordance with the national legislation and the institutional requirements.

## Author Contributions

OA, CG, and AB contributed concept and design of the study. CG organized the database. OA organized the database, analyzed the data, and wrote the first draft of the paper. CG, RM, MG, AA, collected the data. CG, UM-O, AB, PP, and KB read and revised the manuscript. IC analyzed the data and revised the manuscript. All authors contributed to manuscript revision and approved the submitted version.

## Funding

This study is supported by Cancer Center Support Grant 5P30CA056036-17 and the SKCC Biostatistics Shared Resource

## Conflict of Interest

The authors declare that the research was conducted in the absence of any commercial or financial relationships that could be construed as a potential conflict of interest.

## Publisher’s Note

All claims expressed in this article are solely those of the authors and do not necessarily represent those of their affiliated organizations, or those of the publisher, the editors and the reviewers. Any product that may be evaluated in this article, or claim that may be made by its manufacturer, is not guaranteed or endorsed by the publisher.
